# Decentralizing PrEP delivery: Implementation and dissemination strategies to increase PrEP uptake among MSM in Toronto, Canada

**DOI:** 10.1371/journal.pone.0248626

**Published:** 2021-03-18

**Authors:** Maxime Charest, Malika Sharma, Allison Chris, Alexandre Schnubb, David C. Knox, James Wilton, Rita Shahin, Arlene Chan, Sharmistha Mishra, Daniel Grace, Ahmed M. Bayoumi, John Maxwell, Isaac I. Bogoch, Darrell H. S. Tan

**Affiliations:** 1 Ontario Institute for Studies in Education, University of Toronto, Toronto, Ontario, Canada; 2 Division of Infectious Diseases, St. Michael’s Hospital, Toronto, Ontario, Canada; 3 Department of Medicine, University of Toronto, Toronto, Ontario, Canada; 4 Casey House, Toronto, Ontario, Canada; 5 Maple Leaf Medical Clinic, Toronto, Ontario, Canada; 6 Toronto Public Health, Toronto, Ontario, Canada; 7 Centre for Disease Control, University of British Columbia, Vancouver, British Columbia, Canada; 8 Women’s College Hospital, Toronto, Ontario, Canada; 9 MAP Centre for Urban Health Solutions, St. Michael’s Hospital, Toronto, Ontario, Canada; 10 Institute of Medical Science, University of Toronto, Toronto, Ontario, Canada; 11 Institute of Health Policy, Management and Evaluation, University of Toronto, Toronto, Ontario, Canada; 12 Dalla Lana School of Public Health, University of Toronto, Toronto, Ontario, Canada; 13 Division of General Internal Medicine, University of Toronto, Toronto, Ontario, Canada; 14 AIDS Committee of Toronto, Toronto, Ontario, Canada; 15 Division of Infectious Diseases, Toronto General Hospital, Toronto, Ontario, Canada; University of Arkansas for Medical Sciences, UNITED STATES

## Abstract

Pre-exposure prophylaxis (PrEP) is traditionally prescribed by HIV specialist physicians. Given finite specialist resources, there is a need to scale up PrEP delivery by decentralizing services via other healthcare professionals. We aimed to assess the feasibility of delivering PrEP to men who have sex with men (MSM) through primary care physicians and sexual health clinic nurses. We piloted a multi-component, implementation and dissemination research program to increase provision of PrEP through primary care physicians and sexual health clinic nurses in Toronto, Canada. Community-based organizations (CBOs) provided prospective participants with information cards that contained links to an online module on engaging providers in a conversation about PrEP. In our patient-initiated continuing medical education (PICME) strategy, participants saw their family doctors and gave them the card, which also contained a link to a Continuing Medical Education module. In the nurse-led strategy, participants visited one of two participating clinics to obtain PrEP. We administered an optional online questionnaire to patients and providers at baseline and six months. CBOs distributed 3043 cards. At least 339 men accessed the online module and 196 completed baseline questionnaires. Most (55%) intended to visit nurses while 21% intended to consult their physicians. Among 45 men completing follow-up questionnaires at 6 months, 31% reported bringing cards to their physicians and obtaining PrEP through them; sexual health clinics delivered PrEP to 244 patients. Participants who went through the PICME approach reported no changes in relationships with their providers. Nurses showed fidelity to PrEP prescribing guidelines. Nurse-led PrEP and patient-initiated continuing medical education (PICME) for primary care physicians are feasible strategies to increase PrEP uptake. Nurse-led PrEP delivery was preferred by most patients.

## Introduction

Gay, bisexual and other men who have sex with men (MSM) continue to be the population most affected by HIV in Canada, accounting for 41.4% of all incident HIV cases in 2018 [1]. Pre-exposure prophylaxis (PrEP) with the regular use of tenofovir disoproxil fumarate and emtricitabine (TDF/FTC) by uninfected persons at risk of acquiring HIV has shown great effectiveness in reducing HIV incidence. Several population centres, such as London, San Francisco, New South Wales and Vancouver have seen large drops in new HIV diagnoses following widespread access to this intervention [2–5]. In Toronto, Canada, although awareness among MSM has increased steadily since 2012 [6], PrEP rollout has not been systematic.

In Toronto, PrEP has traditionally been prescribed in a ‘centralized’ way, by HIV specialists. However, given its favourable tolerability and toxicity profile, as well as the highly protocolized nature of follow-up, PrEP could readily be provided by primary care providers such as family physicians and sexual health clinic nurses [7]. In one nationwide, Canadian survey of infectious diseases, internal medicine, public health and family medicine physicians, the majority of respondents felt that any doctor should be able to prescribe PrEP, although most did not feel knowledgeable enough to do so [8]. In addition, sexual health nurses, who already provide sexual health counselling, testing and treatment for sexually transmitted infections (STIs), are well-positioned to identify MSM at elevated HIV risk and to provide PrEP.

We therefore developed and piloted two complementary approaches to decentralize PrEP delivery in Toronto: a novel knowledge dissemination strategy which empowered patients themselves to bring information about PrEP to their primary care providers (‘patient-initiated continuing medical education’ or PICME), and a pragmatic implementation strategy in which public health nurses were trained to provide PrEP under a medical directive. Medical directives permit licensed physicians to delegate controlled acts, such as providing medication, to trained personnel, and are already used widely in sexual health clinics. Our primary objective was to quantify the uptake of PrEP achieved among Toronto MSM using each decentralization strategy. Our secondary objectives were to characterize barriers and facilitators to PrEP uptake associated with these strategies, to assess associated changes in the quality of clinician-patient relationships, and to assess fidelity to core components of PrEP delivery.

## Methods

### Program overview

Implementation of our program occurred from September 2017 to December 2019. A detailed description of the methods and procedures can be found elsewhere [9]. In short, a network of community-based organizations (CBOs) who serve a large cross-section of the MSM population in Toronto identified (Step 1) and handed out information cards (Step 2) to potential participants. These cards contained links to two online PrEP modules, one for patients and one for physicians, the latter of which offered CME credits. The patient module (Step 3) educated participants about PrEP and how to engage providers in a conversation about this preventative tool. The physician PrEP module contained practical information about how to prescribe PrEP based on up-to-date, Canadian PrEP guidelines [10], as well as a self-assessment tool. Under the PICME approach, participants would take their cards to their primary care providers (Step 4a) who would complete the physician module (Step 5a), learn about PrEP and ultimately prescribe it to their patients (Step 6a). As an alternative, participants could also book an appointment (Step 4b) to bring their information card into one of two participating Toronto Public Health clinics (Step 5b) where we had trained sexual health nurses to provide PrEP under a medical directive (Step 6b), should any men be unwilling or unable to approach a primary care provider. At the end of each of the modules, patients and providers could complete a baseline questionnaire and were invited to complete a follow-up questionnaire 6 months later. We provided modest compensation for participants who completed these questionnaires.

### Data sources

To achieve our primary objective, we used several data sources to quantify each step in our ‘PrEP decentralization cascade’. To quantify Step 2, we tracked the number of cards we gave to CBOs and subtracted the number that were left at the end of the project. To measure Step 3, we intended to record the number of unique visits to the patient module website. However, we discovered after most of our recruitment was completed that the tracking feature of the website had not been functioning. As such, we instead used the number of post-module, baseline questionnaires that MSM initiated, recognizing that the latter was likely an underestimate of module uptake. Men reported in their baseline questionnaires whether they intended to obtain PrEP through their family physicians or nurses, which we used to estimate an upper bound for Steps 4a/b. We obtained a lower bound for Step 4a/b from the patient 6-month follow-up questionnaires, which asked whether participants had visited their family physician or the sexual health clinic for PrEP, accepting that these would be underestimates if there was attrition between the baseline and follow-up questionnaires. To estimate Step 5a, we used the number of post-CME module baseline questionnaires initiated by clinicians, recognizing that this was likely an underestimate of module uptake. For Step 5b, we asked TPH nurses to estimate the total number of men who came in for a PrEP consultation after implementation of the research program was completed, regardless of whether they came in with an information card or not. Finally, we report the number of men who indicated on their follow-up questionnaires where they had obtained PrEP to estimate the uptake of PrEP through each strategy (Steps 6a/b).

To assess barriers and facilitators of each decentralization approach, quantitative data were drawn from the electronic questionnaires, which included participants’ demographics, sexual activity and substance use, STI history, PEP use, concerns about HIV and attitudes towards PrEP. We also included a validated tool to assess participants’ relationships with their family physicians, addressing physicians’ skills in overall communication (eg. ‘how good is your family doctor at… explaining the results of tests in a way that you understand?’), HIV-related communication (‘…talking with you about your sex life?’), adherence dialogue (‘…giving you information about the right way to take your medications?’) and participatory decision-making (‘…giving you some control over treatment decisions?’), each assessed on a five-point Likert scale [11]. The follow-up questionnaire for MSM also asked participants who used the PICME intervention whether it was associated with changes in the clinician-patient relationship. In addition, we conducted interviews and focus groups with patients, physicians and public health personnel who participated in the study to gain a fuller understanding of the barriers and facilitators of these approaches. These data will be reported on separately.

Finally, to measure fidelity to core components of PrEP delivery, we included questions about how PrEP was prescribed in the physician follow-up questionnaires. These questions asked physicians whether they performed a variety of usual recommended activities throughout the process of prescribing PrEP (e.g. HIV serology, STI and creatinine testing, etc.). However, our low response rate for these surveys precluded analysis for these providers. To measure fidelity among sexual health clinic nurses, we performed chart review at the two sexual health clinics among consenting patients.

### Data analysis

We quantified the PrEP cascade using frequencies and proportions, and used descriptive statistics to characterize participants who completed the baseline questionnaire. Since only a quarter of participants (23%) completed the 6-month follow-up questionnaire, we conducted chi-squares and t-tests, as appropriate, to determine whether this group differed significantly from those who completed the baseline questionnaire.

To assess barriers and facilitators of our PrEP decentralization strategies, we first used logistic regression models to identify factors associated with participants’ intentions to approach their family physician for PrEP, as opposed to the sexual health clinics. This analysis was restricted to participants in the baseline questionnaire who indicated that they had a family doctor and wanted to initiate PrEP (n = 97). The variables that were significant in the univariable models were explored further in three separate multivariable analyses (Models 1, 2 and 3), with selection of covariates based on both clinical reasoning, checks for multicollinearity, and assessment of model fit using the Akaike Information Criterion. We also assessed what proportion of participants who completed the follow-up questionnaire reported improvements, worsening or no change in the quality of their clinician-patient relationships among those who used the PICME approach.

Finally, to assess fidelity to core elements of PrEP prescribing at the sexual health clinics, we quantified the proportion of baseline and follow-up visits for which there was documentation of a) a clinical indication for PrEP (operationalized as condomless anal intercourse with a partner of unknown status in the previous 6 months), b) documentation of a client’s HIV-negative status, c) the correct prescription for no more than 3 months of PrEP with no refills, d) screening for renal toxicity (i.e., creatinine levels), and e) adequate STI screening (i.e., serologic screening for syphilis, as well as pharyngeal, urethral and rectal testing for gonorrhea and chlamydia, as appropriate).

We performed descriptive analyses using SPSS Statistics Version 25.0 and parametric analyses using Stata version 15. We excluded cases with missing values from the analyses.

### Ethics

All study procedures were approved by the St. Michael’s Hospital and Toronto Public Health Research Ethics Boards.

## Results

### PrEP cascade

**Fig 1:**
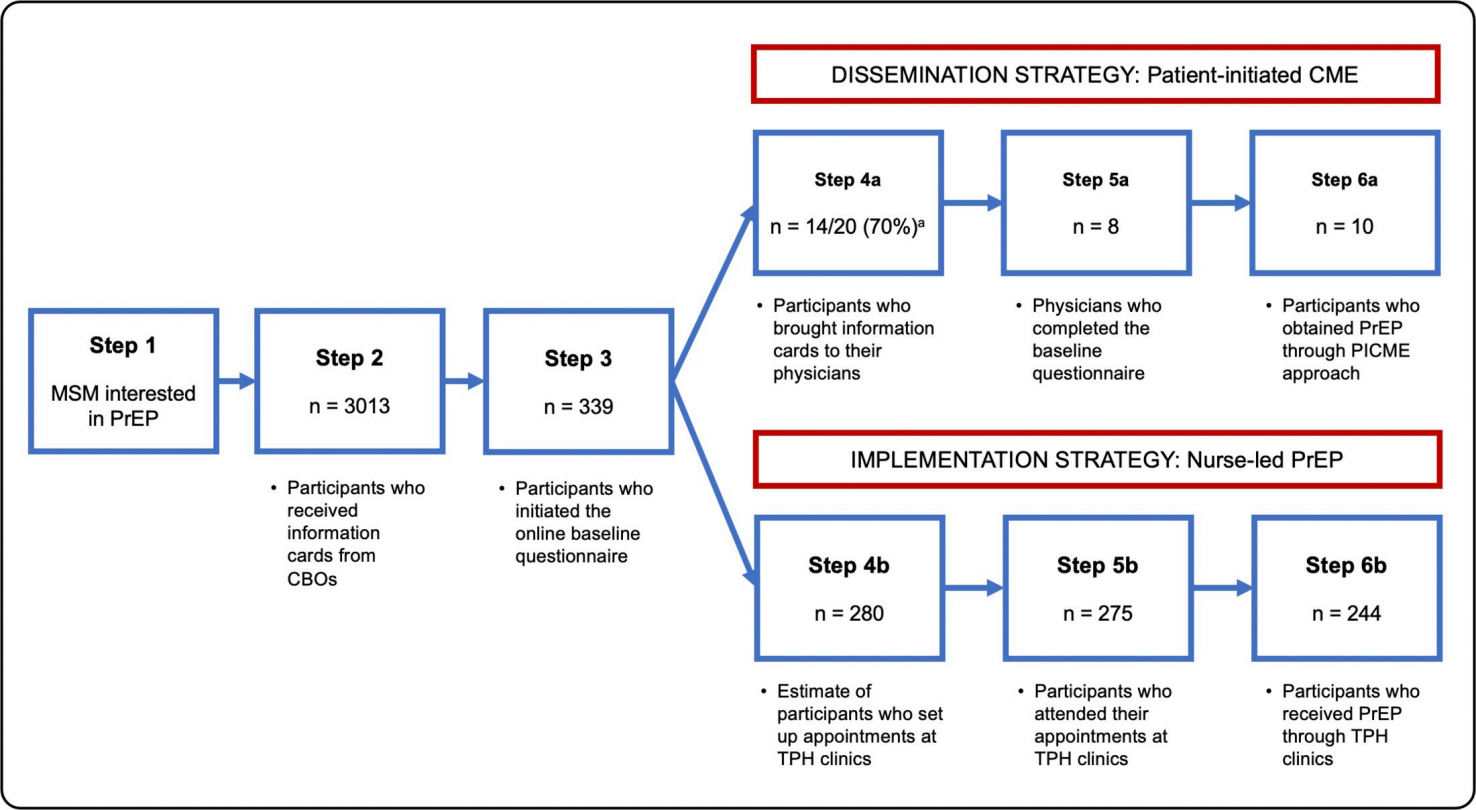
Number of participants who engaged with each step of the PrEP cascade. ^a^ A direct measure for Step 4a was unavailable, hence the number of completed follow-up questionnaires is reported here as the denominator.

In total, CBOs distributed 3013 information cards to prospective participants (Step 2; Fig 1). Of those who received cards, at least 339/3013 (11%) accessed the online module (Step 3), as estimated by the number of individuals initiating the baseline patient questionnaires. Of these, 196/339 (58%) completed the baseline questionnaire. We lacked a direct measure of the number of individuals who actually brought the information card to their primary care providers (Step 4a). However, of 174/196 (89%) participants who answered the question from our baseline patient questionnaire regarding where they intended to obtain PrEP, 41/174 (24%) and 107/174 (62%) reported that they intended to approach their family physicians and STI clinic nurses, respectively. Some men (20/174; 12%) said they would obtain it elsewhere and 6/174 (3%) said they would not initiate PrEP.

In addition, 20/45 (42%) participants who completed the follow-up questionnaire indicated that they had gone to their family doctor after completing the patient module. Of these 20, 14/20 (70%) indicated that they had given the card to that doctor, providing an indirect estimate of Step 4a, and 10/20 (50%) reported that their physician intended to complete the CME module. At least 8 physicians completed the baseline questionnaire and were thus estimated to have completed the CME module (Step 5a). Ultimately, all 10 men who went through the PICME approach (i.e., engaged their providers, gave them a card and whose providers agreed to complete the module) reported receiving PrEP through them. Another 9/20 (45%) who intended to see their provider went to see their physicians, did not give them the card but still initiated PrEP (Step 6a). The total number of physicians who initiated the baseline physician questionnaire was 43, suggesting that the true number of times that a patient used the PICME approach to discuss PrEP with a primary care provider, leading their provider to access the online module, was potentially higher than that estimated in the follow-up patient questionnaires.

Through the nurse-led implementation strategy, a total of 280 patients set up appointments (Step 4b) and an estimated 275/280 (98%) attended them (Step 5b). Ultimately, nurses prescribed PrEP to 244/275 (89%) men (Step 6b). Notably, based on conversations with public health nurses following the program implementation, most men had booked appointments at sexual health clinics after hearing about them through word-of-mouth rather than receiving information cards. However, 24/45 (53%) of those completing the patient follow-up questionnaires reported obtaining PrEP through TPH clinics, compared to 20/45 (42%) who went to see their physicians, suggesting that a slightly higher level of PrEP uptake may have been achieved through the sexual health clinic approach than the PICME approach overall. The remaining 1/45 (2%) obtained PrEP outside of the proposed strategies.

### Demographics

Overall, 196 men completed baseline questionnaires. Participants were aged 18–65 (*Median* = 31.0, *IQR*[26–39.75]) and most identified as male (92.3%) and gay (78.6%). Individuals’ racial and ethnic identities reflected the diversity of the city of Toronto: 53% reported being White, 24% Asian, 7% Latin American, 5% Black and 4% Middle Eastern. A substantial number of men (41%) were not born in Canada. Most participants (76%) had completed some level of postsecondary schooling. Almost half of participants (44%) had an annual, individual income below $60,000 while 25% had an income of $90,000 or more. Further information can be found in Table 1. Only 45 individuals completed the follow-up questionnaire six months later. However, there were no significant differences in age, gender, sexual orientation, education level, race/ethnicity, income and living situation between those who did and did not.

**Table 1:** Demographic characteristics.

Demographic characteristic	Baseline (n = 196)	Follow-Up (n = 45)	*p*-value
Age	33.9 (*SD* = 10.4)	34.1 (*SD* = 10.1)	.97
Gender			
Male	181 (92.3%)	44 (97.8%)	-
Trans (FTM)	1 (0.5%)		
Sexual orientation			
Gay	154 (78.6%)	37 (82.2%)	
Bisexual	21 (10.7%)	6 (13.3%)	
Heterosexual	6 (3.1%)	1 (2.2%)	
Queer	5 (2.6%)		
Other	4 (2.1%)		
Education			
High school diploma	31 (15.8%)	6 (13.3%)	.72
College or undergraduate degree	102 (52.0%)	27 (60.0%)	
Graduate or professional degree	46 (23.5%)	11 (24.4%)	
Race/ethnicity			
White	103 (52.5%)	25 (45.6%)	.41
Asian	48 (24.4%)	13 (28.9%)	
Latin American	13 (6.6%)	4 (8.9%)	
Black	9 (4.6%)		
Middle Eastern	8 (4.3%)	2 (4.4%)	
First Nations	1 (0.5%)		
Indian–Caribbean	1 (0.5%)		
Mixed or Other	6 (3.0%)		
Household income			
$0–29.999	41 (20.9%)	9 (20.0%)	.74
$30,000–59,999	46 (23.5%)	14 (31.1%)	
$60,000–89,999	31 (15.8%)	4 (8.9%)	
$90,000–119,999	18 (9.2%)	5 (11.1%)	
$120,000–149,999	10 (5.1%)	2 (4.4%)	
$150,000 or more	20 (10.2%)	5 (11.1%)	
Prefer not to answer/don’t know	16 (8.1%)	4 (8.9%)	
Housing			
Renting	113 (57.7%)	28 (62.2%)	.32
Own home	59 (30.1%)	15 (33.3%)	
Boarding home	6 (3.1%)	1 (2.2%)	
Supportive housing	3 (1.5%)		
Group home	1 (0.5%)		
Other/prefer not to answer	9 (4.5%)	1 (2.2%)	
Employment			
Student	25 (12.8%)	7 (15.6%)	.41
Part-time	23 (11.7%)	3 (6.7%)	
Full-time	124 (63.3%)	31 (68.9%)	
Not employed	10 (5.1%)	1 (2.2%)	
Born in Canada			
Yes	104 (53.1%)	24 (53.3%)	.76
No	80 (40.8%)	20 (44.4%)	
Prefer not to answer	2 (1.0%)		

*Percentages do not add up to 100% as we did not impute missing data. P-values reflect results from t-tests and chi-squares.

### Secondary outcomes

In univariable logistic regression analyses, factors associated with participants’ intentions on the baseline questionnaire to obtain PrEP through their family physicians, rather than through sexual health clinics, included being ‘out’ to that doctor (*OR* = 10.67, 95%CI = 3.35,33.96), as well as rating their physician as having very good or excellent overall communication skills, (*OR* = 3.42 95%CI = 1.38,8.48), and very good or excellent engagement in participatory decision-making, (*OR* = 3.33 95%CI = 1.14,9.79). These three characteristics were explored further in multivariable Models 1, 2 and 3 (Table 2). All three remained statistically significantly associated with the primary outcome after adjustment for covariates, with adjusted odds ratios (aOR) of 10.17 (95%CI = 2.58, 40.22) for being out to one’s family physician, aOR = 3.37 (95%CI = 1.16,9.84) for very good/excellent physician communication skills, and aOR = 3.97 (95%CI = 1.03, 15.39) for very good/excellent physician skills in participatory decision-making. Only those who, while completing the baseline patient questionnaire, indicated that they had a family physician and intended to obtain PrEP through them were included in this analysis (n = 97).

**Table 2:** Variables associated with intent to seek PrEP from family physicians^a^.

	Univariable models		Model 1		Model 2		Model 3	
Variable	OR (95%CI)	*p*	aOR (95%CI)	*p*	aOR (95%CI)	*p*	aOR (95%CI)	*p*
‘Out’ to family physician^b^	10.67 (3.35, 33.96)	<0.0001	10.17 (2.58, 40.22)	0.0009				
Age	1.03 (0.99, 1.08)	0.13						
Education								
High school	1.00				1.00		1.00	
Any post-secondary	2.80 (0.74, 10.64)	0.13			3.22 (0.61,17.07)	0.35	4.37 (0.80,23.89)	0.09
White race/ethnicity	2.00 (0.79, 5.06)	0.14	1.47 (0.42, 5.09)	0.54				
Income ≥$60,000/year	1.23 (0.53, 2.84)	0.63						
Housing								
Any other housing	1.00							
Owns home	1.07 (0.43,2.67)	0.88						
Employed full-time	2.52 (0.94, 6.77)	0.07						
Canada	0.58 (0.24, 1.40)	0.23						
Any prior bacterial STI	1.58 (0.68, 3.67)	0.28						
Ever used post-exposure prophylaxis	0.40 (0.10, 1.60)	0.20						
Self-perceived lifetime HIV risk moderate/high	1.90 (0.80, 4.55)	0.15						
Frequent (6 monthly or more) HIV/STI testing	0.45 (0.19, 1.06)	0.07						
Usually do HIV/STI testing with family physician	2.49 (0.70, 8.88)	0.16						
HIRI-MSM risk score	0.99 (0.94, 1.05)	0.73	1.01 (0.95, 1.07)	0.81	0.97 (0.91, 1.03)	0.33	0.95 (0.89, 1.02)	0.14
Very good/excellent MD skills in overall communication^b^	3.42 (1.38, 8.48)	0.008			3.37 (1.16, 9.84)	0.03		
Very good/excellent MD skills in HIV-specific information	3.45 (0.77, 15.43)	0.11						
Very good/excellent MD skills in adherence dialogue	1.46 (0.52, 4.06)	0.47						
Very good/excellent MD skills in participatory decision-making^b^	3.33 (1.14, 9.79)	0.03					3.97 (1.03, 15.39)	0.046
Very good/excellent overall satisfaction with MD	2.54 (0.64, 10.19)	0.19						

^a^ Multivariable models are restricted to the n = 97 participants who had a family doctor and declared an intention to start PrEP.

^b^ Primary predictor variable for each model.

Conversely, the content and format of the online physician module itself was likely a facilitator of the PICME approach, in that 10/14 of providers who provided feedback on the module said they would start prescribing PrEP, and of the 8 who provided additional comments, 7 expressed that the program was done well.

Of the 15 men who reported using the PICME approach on their follow-up questionnaires and who answered the question concerning changes in their relationship with their provider, all 15 (100%) said there was no change.

Of the 244 clients who obtained PrEP through sexual health clinic nurses, 93 consented to chart review, with a median (IQR) of 5 (3–6) appointments per person, giving a total of 432 visits overall. Nurses recorded a clinical indication for PrEP (i.e., condomless anal intercourse in the previous 6 months) at 75/93 (81%) baseline and 282/340 (83%) follow-up visits. Laboratory evidence of HIV-negative status was documented and clients underwent creatinine testing (to monitor for renal toxicity) at all visits. Nurses appropriately provided prescriptions for no longer than 3 months at 429/432 (99%) of visits, with no refills. Finally, 84/93 (90%) patients accepted clinically indicated STI testing at baseline visits and 225/340 (66%) at follow-up visits. When nurses did not perform clinically indicated testing, in 65/124 (52%) of cases they recorded that the patients had declined. We further noted that a large proportion of those accessing the sexual health clinics were born outside Canada (57%). The nurses informed us that many of them lacked health insurance, which was an important reason why these men had chosen to access PrEP through these Public Health clinics. In a post-hoc analysis of the patient follow-up questionnaires, we observed that individuals born outside Canada were more likely to have pursued PrEP through the sexual health clinics compared to using the PICME approach (Fisher’s Exact Test *p* = .049).

## Discussion

Engaging a wider array of healthcare providers in PrEP is important for increasing PrEP uptake and decreasing HIV incidence. We evaluated two strategies for decentralizing PrEP delivery to Toronto MSM, by piloting a novel ‘patient-initiated continuing medical education’ (PICME) intervention to engage primary care providers and initiating a nurse-led model of PrEP delivery at two sexual health clinics. While our available data provide challenges in definitively determining how successful these two strategies were in helping interested men obtain PrEP, key findings included preference for PrEP provision via sexual health nurses over family physicians; barriers to seeking PrEP through family physicians including not being ‘out’ to the physician as well as a lack of health insurance; and high fidelity observed with core components of PrEP delivery through the sexual health clinics.

Together, these findings support the further promotion of nurse-led PrEP delivery in sexual health clinics. This model has numerous advantages and represents an attractive form of ‘task-shifting’ that could save costs, facilitate PrEP scale-up, and optimize nurses’ scope of practice [12, 13]. PrEP fits well within public health’s mandate of providing preventative health services and sexual health clinic nurses’ existing skillsets. These nurses are also well-positioned to identify PrEP candidates at high HIV risk, such as those with specific STIs [14–16]. Nurses themselves appear to be in favour of this approach as well; 72.7% of respondents to a survey we recently distributed to nurses in every Ontario sexual health clinic, HIV clinic and community health centre supported nurse-led PrEP [17]. In Ontario, an additional advantage of situating PrEP in sexual health clinics is that they are funded through public health budgets that do not require patients to have health insurance to receive services. As noted above, many of the men who attended public health clinics to obtain PrEP were born outside of the country and did not have health insurance. Because the sexual health clinics did not require clients to have an Ontario health card to receive care, this observation could imply that health insurance may be a barrier in accessing PrEP through family physicians. However, it is important to note that while public health clinics can provide sexual health services to those who are uninsured in our setting, patients still incur the price of the medication. This issue underscores the need to make the medication more financially accessible. Further, our findings show that the use of medical directives can be associated with high fidelity to prescribing guidelines. This evidence is in line with other programs which have shown that nurse-led approaches to preventive HIV care can be very successful [18–21].

However, there may be challenges with the nurse-led model as well. For instance, the longitudinal follow-up of PrEP patients represents a departure from the usual model of episodic care used in most sexual health clinics [22], renal monitoring is unfamiliar to most sexual health nurses [6], and divorcing sexual health from primary care can fragment patients’ healthcare. In Ontario, recent government cuts to public health programs will further jeopardize this approach.

Engaging primary care providers in PrEP delivery thus remains important. Using the implementation science framework outlined by MacLean, Rabin and others, we conceived of our PICME intervention as a knowledge dissemination activity that could address this need, as it employed “an active approach of spreading evidence-based interventions to the target audience via determined channels using planned strategies” [23, 24]. The rationale for our PICME approach was based on several lines of evidence. First, while Canadian guidelines on PrEP delivery were published in 2017, active dissemination is critical to bolster guideline uptake, particularly since PrEP remains unfamiliar to so many clinicians [25]. Recent studies have found that 24% of urban American primary care providers had never heard of PrEP [26], 41% of American primary care providers had only poor or fair knowledge of PrEP [27], and 62.3% family planning providers could not properly define PrEP [28]. Second, literature on physician behaviour shows that willingness to put new interventions into practice is greatest when it is directly requested by patients [29, 30]. Our PICME strategy therefore positioned patients themselves as the agents of knowledge dissemination. Third, we formatted the knowledge as a web-based module because 84.1% of respondents to a survey of Canadian physicians had indicated this as a preferred learning format [8].

Consistent with these design features and with the generally positive evaluations of the CME module itself, the majority of participants who reported giving the information card to their physician indicated that the provider completed the module and ultimately provided a PrEP prescription. Nearly all of the participants who did not provide the information card to their physician still ultimately received PrEP from them. This suggests that additional CME may simply not have been necessary in those cases. The PICME approach could also be applied in other contexts, such as with other populations at risk of acquiring HIV or for those who may have more specialized healthcare needs (e.g., people who inject drugs, transgender people).

Importantly, in addition to strong physician communication and patient engagement skills, the only characteristic associated with willingness to approach a primary care provider rather than a sexual health nurse for PrEP was being ‘out’ to that provider, yet nearly 50% of Canadian MSM have not disclosed their sexual orientation to their family physician [31]. In addition, for men to obtain PrEP through their physicians, they must feel comfortable discussing their sexual behaviours and risks with them [32]. We had postulated that the PICME strategy could open up a dialogue about sexual identity and behaviours with clinicians, but additional interventions are likely needed to help patients feel more comfortable disclosing this information in clinical settings. In our study, perhaps only those patients who were already comfortable discussing their sexual behaviours with their physicians approached them to receive PrEP, given that there was no change in patients’ relationships with their providers. Another potential solution would be a more universal approach to the promotion of PrEP, whereby providers could consistently include PrEP as an option when their patients show interest or when they engage in a conversation with them about sexual health [33]. This could help reduce the limitations imposed by patient self-disclosure of their sexual orientation and sexual practices.

Strengths of our study include the community collaboration involved in module development and participant recruitment, and our use of anonymized, self-completed questionnaires to minimize social desirability bias. A limitation is that we included only MSM; other populations at risk may require different PrEP implementation strategies. In addition, when quantifying our PrEP cascade, we triangulated data from multiple sources due to the impracticality of tracking people individually, our website tracker not functioning, and individuals entering the cascade downstream of Step 1. However, we were still able to infer an overall preference for sexual health clinic nurses over family physicians. There was also high patient attrition between the baseline and follow-up questionnaires, although the characteristics of those lost to follow-up were similar to those retained. Engagement with the program was fairly modest, if we compare the number of participants who accessed the online module to the number of information cards that CBOs distributed (ie. 339 versus 3013). Possible reasons for the low degree of engagement include the self-directed nature of the intervention, and the potential existence of other strategies for accessing PrEP in the community. However, we view this degree of uptake to be reasonable, considering that the intervention only involved receiving an information card from a CBO where PrEP could not be accessed directly. The simplicity of this intervention also lends itself well to replication, perhaps in settings serving at-risk populations where the card or module could be provided directly to patients on a computer or tablet. In addition, there may have been some self-selection bias in terms of the participants who chose to enter into the study (eg. younger age, access to technology, health awareness), which could limit the generalizability of our findings to other populations. Further, a limited number of physicians completed questionnaires, precluding our ability to analyze fidelity in this important stakeholder group. Finally, the TPH PrEP clinics were only open for 3–4 hours/week during working hours, which may have limited accessibility.

## Conclusions

Our findings suggest the feasibility of decentralizing PrEP delivery through sexual health clinic nurses, and provide some evidence for the further study and development of patient-initiated mechanisms of knowledge dissemination. However, ensuring the availability of healthcare providers will not suffice. Large-scale awareness campaigns, universal medication coverage and improved access to related HIV prevention and care services have also been integral to the successful PrEP programs worldwide, and warrant ongoing attention if PrEP is to make a meaningful difference in the epidemic.

## Supporting information

S1 File Patient baseline questionnaire. (PDF)

S2 File Patient follow-up questionnaire. (PDF)

S3 File Physician baseline questionnaire. (PDF)

S4 File Physician follow-up questionnaire. (PDF)
