# Supplementary material for: Decentralizing PrEP delivery: Implementation and dissemination strategies to increase PrEP uptake among MSM in Toronto, Canada
Source: PLoS One. 2021 Mar 18;16(3):e0248626. doi: 10.1371/journal.pone.0248626 (PMC7971529; doi:10.1371/journal.pone.0248626)
Supplement: S1 File — (PDF) [file pone.0248626.s001.pdf]

APPENDIX E: Decentralizing PrEP Delivery Patient Baseline Questionnaire

INSTRUCTIONS: Please fill out this questionnaire to the best of your ability. Thank you very much for taking the time to participate in this study. The first few questions are about you.

1. Please enter the unique ID code on the paper or e-card you used to access this module: ______

2. How old are you? __________

3. How do you identify?

   - ☐ Male 1
   - ☐ Female 2
   - ☐ Male to female trans 3
   - ☐ Female to male trans 4
   - ☐ Intersex 5
   - ☐ Other (specify): ________6
   - ☐ Prefer not to answer

4. What is the highest level of education you have completed?

   - ☐ Less than a high school diploma 1
   - ☐ High school diploma 2
   - ☐ College or undergraduate degree 3
   - ☐ Graduate or Professional degree 4

5. Which of the following best describes your racial or ethnic group? CHECK ONE ONLY.

- ☐ Asian - East (e.g., Chinese, Japanese, Korean) 3
- ☐ Asian - South (e.g., Indian, Pakistani, Sri Lankan) 4
- ☐ Asian - South East (e.g., Malaysian, Filipino, Vietnamese) 5
- ☐ Black - African (e.g., Ghanaian, Kenyan, Somali) 8
- ☐ Black - Caribbean (e.g., Barbadian, Jamaican) 9
- ☐ Black - North American (e.g., Canadian, American) 7
- ☐ First Nations 12
- ☐ Indigenous/Aboriginal not included elsewhere
- ☐ Indian – Caribbean (e.g., Guyanese with origins in India) 13
- ☐ Inuit
- ☐ Latin American (e.g., Argentinean, Chilean, Salvadorian) 6
- ☐ Métis
- ☐ Middle Eastern (e.g., Egyptian, Iranian, Lebanese) 10
- ☐ White - European (e.g., English, Italian, Portuguese, Russian) 2
- ☐ White - North American (e.g., Canadian, American) 1
- ☐ Mixed heritage (e.g., Black- African and White-North American) (Please specify) 11 _________________________________
- ☐ Other(s) (Please specify) _____________
- ☐ Prefer not to answer
- ☐ Do not know

6. What language would you feel most comfortable speaking in with your healthcare provider?

- English 1
- French 2
- Other 3

Pop up if other:

- Amharic
- Arabic
- ASL
- Bengali
- Chinese (Cantonese)
- Chinese (Mandarin)
- Czech
- Dari
- Farsi
- Greek
- Hindi
- Hungarian
- Italian
- Karen
- Korean
- Nepali
- Polish
- Portuguese
- Punjabi
- Russian
- Serbian
- Slovak
- Somali
- Spanish
- Tagalog
- Tamil
- Tigrinya
- Turkish
- Twi
- Ukrainian
- Urdu
- Vietnamese
- Prefer not to answer
- Do not know
- Other (Please specify) _______________

7. What was your total household income before taxes last year? CHECK ONE ONLY.

- $0 to $29,999 1
- $30,000 to $59,999 2
- $60,000 to $89,999 3
- $90,000 to $119,999 4
- $120,000 to $149,999 5
- $150,000 or more 6
- Prefer not to answer 7
- Do not know 8

8. What type of housing do you live in? CHECK ONE ONLY.

- Boarding Home 4
- Correctional Facility
- Do not know
- Group Home 5
- Homeless/on street
- Couch surfing
- Own home 2
- Renting 1
- Shelter/Hostel
- Supportive Housing 3
- Other (Please specify) ______
- Prefer not to answer 6

9. Are you currently employed?

- ☐ Full Time 3
- ☐ Student 1
- ☐ Part Time 2
- ☐ Not employed 4

10. Were you born in Canada?

- ☐ Yes 1
- ☐ No 0
- ☐ Prefer not to answer 2
- ☐ (Popup) If NO, what year did you arrive in Canada ________

11. How would you describe your sexual orientation?

- ☐ Gay 1
- ☐ Bisexual 2
- ☐ Heterosexual 4
- ☐ Queer 3
- ☐ Two-spirited
- ☐ Do not know
- ☐ Prefer not to answer
- ☐ Other (specify): ________________________

The next few questions are about sexual activity during the past six months. Please write the number of times you did these activities. If a question does not apply to you, please write Not Applicable (N/A).

12. How many MEN have you had sex with in the last 6 months? __________________
13. Among those Y (pop up of number entered in 12) men, how many did you know were HIV-positive? __
14. Among those Y men, how many were of unknown HIV status? ___________

15. How many **TIMES** in the past 6 months have you participated in the following sexual activities? (Please use **NUMBERS ONLY** in this space)

		**Number of TIMES with an HIV positive partner**	**Number of TIMES with an HIV negative partner**	**Number of TIMES with a partner whose HIV status I didn't know**
**a**	Receptive anal sex (you were the bottom) with a man **WITHOUT** a condom			
**b**	Receptive anal sex (you were the bottom) with a man **WITH** a condom			
**c**	Insertive anal sex (you were the top) with a man **WITHOUT** a condom			
**d**	Insertive anal sex (you were the top) with a man **WITH** a condom			

**e**	Vaginal intercourse **WITHOUT** a condom			
**f**	Vaginal intercourse **WITH** a condom			

16. In the past 6 months, which of the following have you used at least once? Check all that apply. 0 = No, 1 = Yes, 2 = N/A

- ☐ Alcohol
- ☐ Cocaine (smoking/snorting)
- ☐ Crack cocaine (smoking/snorting)
- ☐ Injectable drugs (heroin, crystal, etc.)
- ☐ Prescription opioids
- ☐ Other recreational drugs
- ☐ None
- ☐ Marijuana (Weed)
- ☐ Methamphetamines (non-injection crystal, speed, etc)
- ☐ Poppers (Amyl Nitrate)

17. Have you ever been diagnosed with any of these sexually transmitted infections (STIs)? Check all that apply. 0 = No, 1 = Yes, 2 = N/A

- ☐ Chlamydia or LGV- rectal (anus, ass, bum)
- ☐ Chlamydia or LGV-other (throat, penis)
- ☐ Chlamydia- unsure of type
- ☐ Genital herpes
- ☐ Genital warts
- ☐ Gonorrhea- rectal (anus, ass, bum)
- ☐ Gonorrhea-other (throat, penis)
- ☐ Gonorrhea-unsure of type
- ☐ Syphilis
- ☐ Never had an STI
- ☐ I am not sure

18. Post-exposure prophylaxis (PEP) is the use of anti-HIV medications AFTER sexual activity or injection drug use to prevent HIV. PEP is taken as oral pills daily for 28 days. Have you ever taken POST-exposure prophylaxis after sexual activity to prevent HIV?

- ☐ Yes = 1
- ☐ No = 0
- ☐ Unsure/cannot recall

The next few questions are about your potential concerns about HIV.

19. What do you think your risk of getting HIV **IN YOUR LIFETIME** is?

- ☐ No risk 4
- ☐ A little bit of risk (low risk) 3
- ☐ More than a little bit of risk (moderate risk) 2
- ☐ A lot of risk (high risk) 1

19a POP UP: How would you quantify that risk, on a scale from 0 to 100%?

19b (pop up) If you have indicated "no risk" or "a little bit of risk (low risk)," please indicate why you feel this way. Check all that apply. 0 = No, 1 = Yes, 2 = N/A

- ☐ Low number of partners
- ☐ I believe/know my partner(s) are HIV-negative
- ☐ I believe/know my HIV-positive partner(s) have an undetectable viral load
- ☐ I usually use condoms
- ☐ I am on PrEP and take it regularly
- ☐ I am usually the top (insertive partner) for anal sex
- ☐ Other, please specify: ________________________________

20. How likely do you think you are to get HIV **IN THE NEXT YEAR**, on a scale from 0 to 100%? _____%

21. How concerned are you with your current level of HIV risk?

- ☐ Not concerned at all = 4
- ☐ A little bit concerned = 3
- ☐ More than a little bit concerned = 2
- ☐ Very concerned = 1

22. **Please rate how strongly you agree or disagree with the following statements about HIV (1=strongly disagree, 5 = strongly agree).**

	1. Strongly Disagree	2. Disagree	3. Neutral	4. Agree	5. Strongly Agree
a) Since the discovery of new drugs to fight HIV, I'm less concerned about getting infected.					
b) Now that HIV is a manageable disease, I feel more free to have sex without condoms.					
c) With all the talk of new HIV medicines, AIDS doesn't seem so scary anymore.					

d) I'm less concerned about getting AIDS now that there are new effective medicines to treat it.					
e) The new antiviral drugs are making it less scary to get AIDS.					
f) I wouldn't be so upset if I got infected with HIV because there are powerful new drugs to treat it.					
g) I've taken chances I regretted the next morning.					
h) I give myself grief about not protecting myself.					
i) When I get a cold/flu I start worrying that I got infected with HIV.					

The next few questions are about HIV Pre-exposure prophylaxis.

Pre-exposure prophylaxis (PrEP) is a new strategy for HIV prevention. It involves the use of a prescription pill on a daily basis by a person who is HIV-negative, in order to reduce their risk of becoming infected with HIV. It is taken on an ongoing basis, both before and after an exposure, for potentially as long as a person is at risk of becoming infected with HIV. The pill contains two antiretroviral drugs (tenofovir/emtricitabine) combined into a single pill and is known by the name Truvada®. Research suggests that it is generally safe and is over 90% effective if taken consistently. It is much less effective if not taken as directed and it does not protect against other STIs. Taking PrEP would require a visit to a doctor every 3 months in order to be tested for HIV, STIs and side effects. Truvada® has been approved for use as PrEP in Canada.

23. Please indicate to what extent you agree with the following statement: "I am interested in taking PrEP to reduce my current risk of HIV infection."

- ☐ 1 Strongly disagree
- ☐ 2 Disagree
- ☐ 3 Neutral
- ☐ 4 Agree
- ☐ 5 Strongly Agree

23a POPUP If you answered disagree or strongly disagree to question 23, please indicate why you are currently not interested in PrEP. Check all that apply. 0 = No, 1 = Yes, 2 = N/A

- I am not high risk enough
- I am concerned about side-effects
- It is not 100% effective
- I don’t trust the science
- I don’t want to visit a doctor every 3 months
- I am worried what people would think of me
- Other, please specify: ______________________________

23b POPUP If you answered agree or strongly agree to question 23, please indicate all the reasons you are not currently taking PrEP. Check all that apply. 0 = No, 1 = Yes, 2 = N/A

- I don’t have a doctor
- I haven’t felt comfortable asking my doctor about PrEP
- I asked my doctor about PrEP but they wouldn’t prescribe it
- I have been meaning to, but I haven’t got around to it yet
- I don’t have private health insurance
- I don’t think my private health insurance covers the cost
- I know my private health insurance does not cover the cost
- I didn’t know about it before
- Other, please specify: ________________________________

24. Please indicate your main reasons for being interested in PrEP. Check all that apply. 0 = No, 1 = Yes, 2 = N/A
    - To lower my risk of HIV
    - To lower my anxiety about getting HIV
    - To increase sexual pleasure by having condomless sex
    - To increase intimacy by having condomless sex
    - My partner(s) are pressuring me to go on PrEP
    - I'm not interested in PrEP
    - Other, please specify: _________________________

25. How often do you usually get tested for HIV/STIs?
    - I have never had HIV/STI testing 6
    - I have only had HIV/STI testing once before 5
    - I get tested less than once a year 4
    - I get tested once a year 3
    - I get tested every 6 months 2
    - I get tested every 3 months 1

25a POPUP if >once prior testing: Where do you usually get tested for HIV/STIs?

- I usually do HIV/STI my testing with my family doctor 3
- I usually do HIV/STI my testing at walk-in clinics 4
- I usually do HIV/STI my testing at sexual health clinics 1

- I do HIV/STI testing at a mix of different locations 2

The last few questions are about your family doctor.

26. Do you currently have a family doctor? 0 = No, 1 = Yes, 2 = N/A

- Yes (if yes, sends to Q 27)
- No (if no, sends to end of survey)

27. Are you “out” to your family doctor? 0 = No, 1 = Yes, 2 = N/A

- Yes
- No

27a POPUP if 27=no What are some of the reasons you are not “out” to your family doctor? Check all that apply 0 = No, 1 = Yes, 2 = N/A

- The topic has never come up
- I am not out to very many people at all
- I do not think my family doctor is knowledgeable about issues related to sexual orientation
- I do not think my family doctor is comfortable discussing issues related to sexual orientation
- I think my family doctor is homophobic
- Other (specify)

28. For the next question, please rate your family doctor in each of the following items. How is your family doctor at:

	1. Poor	2. Fair	3. Good	4. Very Good	5. Excellent
a) Explaining the results of tests in a way that you understand?					
b) Giving you facts about the benefits and risks of treatment?					
c) Telling you what to do if certain problems or symptoms occur?					
d) Demonstrating caring, compassion and understanding?					
e) Understanding your health worries and concerns?					
f) Talking with you about your sex life?					
g) Asking you about stresses in your life that may affect your health?					

h) Asking about problems with alcohol?					
i) Asking about problems with street drugs like cocaine or heroin?					
j) Giving you information about the right way to take your medications?					
k) Understanding the problems you have taking your medications?					
l) Helping you solve problems you have taking your medications the right way?					

29. If there was a choice between treatments, would your family doctor ask you to help make the decision?

- ☐ Definitely yes 5
- ☐ Probably yes 4
- ☐ Uncertain 3
- ☐ Probably not 2
- ☐ Definitely not 1

30. How often does your family doctor do the following things:

	1. None of the time	2. A little of the time	3. Sometimes	4. Most of the time	5. All of the time
a) ask you to take some of the responsibility of your treatment (i.e medication, quitting smoking)?					
b) make an effort to give you some control over your treatment?					

31. Which of the following best describes your intentions regarding PrEP?

- ☐ I plan to ask my family doctor to start me on PrEP 2
- ☐ I plan to ask a nurse at one of the Toronto Public Health sexual clinics indicated in this study to start me on PrEP 1
- ☐ I do not plan on starting PrEP 3
- ☐ Other
- ☐

32. Please enter your email address here. We will retain your email address and unique ID code in an electronic file under password protection on a secure server behind a firewall. Your email address will only be used to

contact you about a follow-up survey, and in case you need to contact us to recall your unique ID code. This email address will also be used to email you your $20 electronic gift card.

Thank you for taking the time to complete our survey. We appreciate your time.

If you have further questions regarding HIV prevention, please contact your doctor or call the Canadian AIDS Treatment Information Exchange. CATIE is Canada's source for up-to-date, unbiased information about HIV and hepatitis C. CATIE connects people living with HIV or hepatitis C, at-risk communities, healthcare providers and community organizations with knowledge, resources and expertise to reduced transmission and improve quality of life. For more details, please visit www.catie.ca or call 1-800-263-1638.

If you do not have a family doctor, you can access HealthCare Connect to look for one here: https://www.ontario.ca/page/find-family-doctor-or-nurse-practitioner
