# Supplementary material for: Decentralizing PrEP delivery: Implementation and dissemination strategies to increase PrEP uptake among MSM in Toronto, Canada
Source: PLoS One. 2021 Mar 18;16(3):e0248626. doi: 10.1371/journal.pone.0248626 (PMC7971529; doi:10.1371/journal.pone.0248626)
Supplement: S2 File — (PDF) [file pone.0248626.s002.pdf]

APPENDIX F: Decentralizing PrEP Delivery Patient Followup Questionnaire

INSTRUCTIONS: Please fill out this questionnaire to the best of your ability. Thank you very much for taking the time to participate in this study. The first few questions are about you.

1. Please enter the unique ID code on the paper or e-card you used to access this module: ______

The next few questions are about sexual activity during the past six months. Please write the number of times you did these activities. If a question does not apply to you, please write Not Applicable (N/A).

2. How many MEN have you had sex with in the last 6 months? _________________
3. How many of those men did you know were HIV-positive? _____________
4. How many of those men were of unknown HIV status? __________

5. How many times in the past 6 months have you participated in the following sexual activities?

(Please use **NUMBERS ONLY** in this space)

		**Number of times with an HIV positive partner**	**Number of times with an HIV negative partner**	**Number of times with a partner whose HIV status I didn't know**
**a)**	Receptive anal sex (you were the bottom) with a man **WITHOUT** a condom			
**b)**	Receptive anal sex (you were the bottom) with a man **WITH** a condom			
**c)**	Insertive anal sex (you were the top) with a man **WITHOUT** a condom			
**d)**	Insertive anal sex (you were the top) with a man **WITH** a condom			
**e)**	Vaginal intercourse **WITHOUT** a condom			
**f)**	Vaginal intercourse **WITH** a condom			

6. In the past 6 months, which of the following have you used? Check all that apply. 0 = No, 1 = Yes, 2 = N/A

- ☐ Alcohol
- ☐ Cocaine (smoking/snorting)
- ☐ Crack cocaine (smoking/snorting)
- ☐ Injectable drugs (heroin, crystal, etc.)
- ☐ Prescription opioids
- ☐ Marijuana (Weed)

- ☐ Methamphetamines (non-injection crystal, speed, etc)
- ☐ Poppers (Amyl Nitrate)
- ☐ Other recreational drugs
- ☐ None

7. In the last six months, have you been diagnosed with any of these sexually transmitted infections (STIs)?

   Check all that apply. 0 = No, Yes = 1, 2 = N/A

   - ☐ Chlamydia or LGV- rectal (anus, ass, bum)
   - ☐ Chlamydia or LGV-other (throat, penis)
   - ☐ Chlamydia- unsure of type
   - ☐ Genital herpes
   - ☐ Genital warts
   - ☐ Gonorrhea- rectal (anus, ass, bum)
   - ☐ Gonorrhea-other (throat, penis)
   - ☐ Gonorrhea-unsure of type
   - ☐ Syphilis
   - ☐ Never had an STI
   - ☐ I am not sure

The next few questions are about your potential concerns about HIV.

8. What do you think your risk of getting HIV **IN YOUR LIFETIME** is?

   - ☐ No risk 1
   - ☐ A little bit of risk (low risk) 2
   - ☐ More than a little bit of risk (moderate risk) 3
   - ☐ A lot of risk (high risk) 4

8a. POPUP how would you quantify that risk, on a scale from 0-100%? ____

8a: POPUP If you have indicated "no risk" or "a little bit of risk (low risk)," please indicate why you feel this way. Check all that apply. 0 = No, 1 = Yes, 2 = N/A

- ☐ Low number of partners
- ☐ I believe/know my partner(s) are HIV-negative
- ☐ I believe/know my HIV-positive partner(s) have an undetectable viral load
- ☐ I usually use condoms
- ☐ I am on PrEP and take it regularly
- ☐ I am usually the top (insertive partner) for anal sex
- ☐ Other, please specify: ________________________________

9. How likely do you think you are to get HIV **IN THE NEXT YEAR**, on a scale from 0 to 100%? ___

10. How concerned are you with your current level of HIV risk?

    - ☐ Not concerned at all 1
    - ☐ A little bit concerned 2
    - ☐ More than a little bit concerned 3
    - ☐ Very concerned 4

The next few questions are about HIV Pre-exposure prophylaxis.

Pre-exposure prophylaxis (PrEP) is a new strategy for HIV prevention. It involves the use of a prescription pill on a daily basis by a person who is HIV-negative, in order to reduce their risk of becoming infected with HIV. It is taken on an ongoing basis, both before and after an exposure, for potentially as long as a person is at risk of becoming infected with HIV. The pill contains two antiretroviral drugs (tenofovir/emtricitabine) combined into a single pill and is known by the name Truvada. Research suggests that it is generally safe and is over 90% effective if taken consistently. It is much less effective if not taken as directed and it does not protect against other STIs. Taking PrEP would require a visit to a doctor every 3 months in order to be tested for HIV, STIs and side effects. Truvada® has been approved for use as PrEP in Canada.

11. Please indicate to what extent you agree with the following statement: "I am interested in taking PrEP to reduce my current risk of HIV infection."

- ☐ Strongly disagree 1
- ☐ Disagree 2
- ☐ Neutral 3
- ☐ Agree 4
- ☐ Strongly Agree 5

11a POPUP If you answered disagree or strongly disagree to question 11, please indicate why you are not interested in PrEP. Check all that apply. 0 = No, 1 = Yes, 2 = N/A

- ☐ I am not high risk enough
- ☐ I am concerned about side-effects
- ☐ It is not 100% effective
- ☐ I don't trust the science
- ☐ I don't want to visit a doctor every 3 months
- ☐ I am worried what people would think of me
- ☐ Other, please specify: ________________________________

11b If you answered strongly agree or agree to Q 11, please indicate your main reasons for still being interested in PrEP. Check all that apply. 0 = No, 1 = Yes, 2 = N/A

- ☐ To decrease my risk of HIV
- ☐ To decrease my anxiety about getting HIV
- ☐ To increase sexual pleasure by having condomless sex
- ☐ To increase intimacy by having condomless sex
- ☐ My partner(s) are pressuring me to go on PrEP
- ☐ I'm not interested in PrEP
- ☐ Other, please specify: _________________________________

11c If you answered strongly agree or agree to Q 11, in which setting would you most prefer to do your PrEP-related checkups?

- ☐ A family doctor 2
- ☐ A sexual health clinic 1
- ☐ A hospital specialist 3
- ☐ I'm not interested in PrEP 4
- ☐ Other, please specify: ________________________________

12. Did you complete the module about PrEP? 2 = N/A
    - ☐ Yes 1
    - ☐ No 0

13. After completing the module, did you go see your family doctor about PrEP? 2 = N/A
    - ☐ Yes (if yes, jumps to Q14) 1
    - ☐ No (if no, jumps to Q 16) 0

13a If 13=Yes: Did you give your family doctor the card to access education about PrEP? 2 = N/A

- ☐ Yes 1
- ☐ No 0

14. Did your family doctor agree to do the education module about PrEP? 2 = N/A
    - ☐ Yes 1
    - ☐ No 0

15. Did you see your family doctor at a 2nd or follow up appointment to talk more about PrEP? 2 = N/A
    - ☐ Yes 1
    - ☐ No 0

16. Did you see a Toronto Public Health Nurse about PrEP? 2 = N/A
    - ☐ Yes 1
    - ☐ No 0

17. Did you get a prescription for PrEP? 2 = N/A
    - ☐ Yes 1
    - ☐ No 0

18. How useful do you feel the module was in helping you discuss PrEP with your healthcare provider.
    - ☐ Extremely helpful 5
    - ☐ Very helpful 4
    - ☐ Moderately helpful 3
    - ☐ Slightly helpful 2
    - ☐ Not at all helpful 1

19. How comfortable were you discussing your sexual practices with your healthcare provider?
    - ☐ Very uncomfortable 1
    - ☐ Uncomfortable 2
    - ☐ Neutral 3
    - ☐ Comfortable 4

- ☐ Very comfortable 5

20. Which of the following issues did your healthcare provider discuss with you at your initial visit (BEFORE they completed their module)? Check all that apply. 0 = No, 1 = Yes, 2 = N/A
    - ☐ Sexual behaviours (oral sex, anal sex)
    - ☐ Condom use
    - ☐ Recreational drug use
    - ☐ Alcohol use
    - ☐ Adherence (ability to take medications regularly)
    - ☐ Cost/drug insurance
    - ☐ Other sexually transmitted infections (e.g. gonorrhea, chlamydia, syphilis)

21. Which of the following issues did your healthcare provider discuss with you at your second or a follow-up visit (AFTER they would have completed their module)? Check all that apply. 0 = No, 1 = Yes, 2 = N/A
    - ☐ Sexual behaviours (oral sex, anal sex)
    - ☐ Condom use
    - ☐ Recreational drug use
    - ☐ Alcohol use
    - ☐ Adherence (ability to take medications regularly)
    - ☐ Cost/drug insurance
    - ☐ Other sexually transmitted infections (e.g. gonorrhea, chlamydia, syphilis)

22. Do you feel as though your healthcare provider was able to answer your questions about PrEP after they went through the continuing medical education module? 2 = N/A
    - ☐ Yes 1
    - ☐ No 0

The last few questions are about your family doctor.

23. Do you have a family doctor? 2 = N/A
    - ☐ Yes (if yes, go to 24) 1
    - ☐ No (if no, go to end of survey) 0

24. How has your experience providing the information card and discussing PrEP with your doctor (if applicable) impacted the quality of your doctor-patient relationship?
    - ☐ Worsened the relationship a great deal 1
    - ☐ Worsened the relationship somewhat 2
    - ☐ No change 3
    - ☐ Improved the relationship somewhat 4
    - ☐ Improved the relationship a great deal 5

24a POPUP Can you describe how your relationship with your doctor has changed? (Free text)

25. Are you now “out” to your family doctor? 2 = N/A

- ☐ Yes 1
- ☐ No 0

25a) POPUP if no What are some of the reasons you are not “out” to your family doctor? Check all that apply
0 = No, 1 = Yes, 2 = N/A

- ☐ The topic has never come up
- ☐ I am not out to very many people at all
- ☐ I do not think my family doctor is knowledgeable about issues related to sexual orientation
- ☐ I do not think my family doctor is comfortable discussing issues related to sexual orientation
- ☐ I think my family doctor is homophobic
- ☐ Other (specify)

26. For the next question, please rate your family doctor in each of the following items. How is your family doctor at:

	1. Poor	2. Fair	3. Good	4. Very Good	5. Excellent
a) Explaining the results of tests in a way that you understand?					
b) Giving you facts about the benefits and risks of treatment?					
c) Telling you what to do if certain problems or symptoms occur?					
d) Demonstrating caring, compassion and understanding?					
e) Understanding your health worries and concerns?					
f) Talking with you about your sex life?					
g) Asking you about stresses in your life that may affect your health?					
h) Asking about problems with alcohol?					
i) Asking about problems with street drugs like cocaine or heroin?					
j) Giving you information about the right way to take your medications?					
k) Understanding the problems you have taking your medications?					

l) Helping you solve problems you have taking your medications the right way?					

27. If there was a choice between treatments, would your family doctor ask you to help make the decision?

- ☐ Definitely yes 5
- ☐ Probably yes 4
- ☐ Uncertain 3
- ☐ Probably not 2
- ☐ Definitely not 1

28. How often does your family doctor do the following things:

	1. None of the time	2. A little of the time	3. Sometimes	4. Most of the time	5. All of the time
a) ask you to take some of the responsibility of your treatment?					
b) make an effort to give you some control over your treatment?					

Thank you for taking the time to complete our survey. We appreciate your time.

If you have further questions regarding HIV prevention, please contact your doctor or call the Canadian AIDS Treatment Information Exchange. CATIE is Canada's source for up-to-date, unbiased information about HIV and hepatitis C. CATIE connects people living with HIV or hepatitis C, at-risk communities, healthcare providers and community organizations with knowledge, resources and expertise to reduced transmission and improve quality of life. For more details, please visit www.catie.ca or call 1-800-263-1638.

