# Supplementary material for: Decentralizing PrEP delivery: Implementation and dissemination strategies to increase PrEP uptake among MSM in Toronto, Canada
Source: PLoS One. 2021 Mar 18;16(3):e0248626. doi: 10.1371/journal.pone.0248626 (PMC7971529; doi:10.1371/journal.pone.0248626)
Supplement: S3 File — (PDF) [file pone.0248626.s003.pdf]

APPENDIX H: Decentralizing PrEP Delivery Physician Baseline Questionnaire

INSTRUCTIONS: Please fill out this questionnaire to the best of your ability. Thank you very much for taking the time to participate in this study. The first few questions are about you and your practice.

1. Please enter the unique ID code on the paper or e-card you used to access this module: ______

2. What are the first three characters of your practice's postal code?
   □□□

3. Do you identify as:

- □ Male
- □ Female
- □ Trans Man (F to M)
- □ Trans Woman (M to F)
- □ None of these describe me (specify) ___________________________

4. In what type of setting do you practice predominantly?

- □ Private practice
- □ Community hospital
- □ Academic hospital
- □ Community health centre
- □ Walk-in clinic
- □ Sexual health clinic
- □ Other, please specify: ___________________________

5. What year did you complete medical school?

6. Do you identify as a specialist in HIV care?
- □ Yes
- □ No

7. What proportion of your patient population is persons living with HIV?
   - □ 0-10%
   - □ 11-20%

- ☐ 21-30%
- ☐ 31-40%
- ☐ 41-50%
- ☐ 51-60%
- ☐ 61-70%
- ☐ 71-80%
- ☐ >81%

8. What proportion of your patient population is HIV-negative but at high risk for HIV acquisition (please enter an estimated percentage)? _____

9. How many hours a week do you spend providing direct patient care? _____

The next few questions are about HIV Pre-exposure Prophylaxis, or PrEP.

PrEP is a new, Health Canada-approved, evidence-based HIV prevention method that involves an HIV-uninfected individual taking specific antiretroviral drugs to reduce their risk of becoming infected with HIV. Typically, PrEP is prescribed as one tablet of tenofovir disoproxil fumarate/emtricitabine 200/300 mg daily, and can reduce HIV risk by >90% among men who have sex with men if adherence is high. Individuals taking PrEP require regular testing for HIV and other sexually transmitted infections as well as plasma creatinine. PrEP is NOT the same as post-exposure prophylaxis or PEP, which is the daily use of antiretroviral pills for 28 days AFTER a potential exposure.

10. How would you describe your current knowledge about PrEP?
    - ☐ Not familiar at all (eg. this patient encounter was my first time hearing about it)
    - ☐ Somewhat familiar (eg. I am aware of PrEP and the existence of clinical trials but not of their details)
    - ☐ Very familiar (eg. I am aware of the details of recent clinical trials)

11. How would you rate your current level of comfort prescribing PrEP?

    - ☐ 1 Not comfortable at all
    - ☐ 2
    - ☐ 3 Somewhat comfortable
    - ☐ 4
    - ☐ 5 Very comfortable

12. Have you personally ever prescribed PrEP to a patient?

    - ☐ Yes
    - ☐ No

- Unsure/cannot recall

12a. POPUP if yes: To how many different patients have you prescribed PrEP in the past 12 months?

13. Prior to being asked by the patient who gave you the card to access this CME module, has a patient ever asked you about PrEP?

- Yes
- No
- Unsure/cannot recall

13a POPUP if yes: How many different patients have asked you about PrEP in the past 12 months? __

14. Do you have any patients in your practice that are being prescribed PrEP by another provider (eg. HIV specialist)?

Yes

No

Unsure

14a if 14=yes: How many? ____

15. Regarding the patient who gave you the card to access this CME module – how long have they been a patient under your care?

- It was our first visit
- <1 year
- _____ years

15. How would you describe the quality of your doctor-patient relationship with this patient?
    - Poor – our interactions are tense, awkward, or feel not entirely honest
    - Fair – we have some challenges in communication
    - Average
    - Good – my patient appears to trust me and our interactions feel positive
    - Excellent – we have a trusting and collaborative relationship

16. Based on your current experience and understanding of PrEP, what are your thoughts on PrEP as an HIV prevention method for your patients? Based on what you know currently, is your attitude towards PrEP generally:

- Very Unenthusiastic – I do not feel PrEP is an appropriate means of preventing HIV
- Unenthusiastic

- ☐ Neutral – I feel neutral about the utility of PrEP for HIV prevention
- ☐ Enthusiastic
- ☐ Very enthusiastic- I think PrEP is an essential tool in HIV prevention and am eager to prescribe it to appropriate patients.

17. The next few questions are about HIV more broadly. Below is a list of ideas about HIV+ patients. Some of the ideas may be true for you, and some of them may not. People hold a wide range of ideas about HIV+ patients, and we are interested in your particular ideas. Please answer the questions honestly – your responses are completely de-identified.

	Strongly Disagree	Disagree	Somewhat disagree	Somewhat Agree	Agree	Strongly Agree
a) I believe most HIV+ patients acquired the virus through risky behaviour.						
b) I think HIV+ patients have engaged in risky activities despite knowing these risks.						
c) I believe I have the right to refuse to treat HIV+ patients for the safety of other patients.						
d) I think people would not get HIV if they had sex with fewer people.						
e) HIV+ patients present a threat to my health.						
f) HIV+ patients present a threat to the health of other patients.						
g) I believe I have the right to refuse to treat HIV+ patients if other staff members are concerned about safety.						
h) I would avoid conducting certain procedures on HIV+ patients.						
i) I think if people act responsibly they will not						

contract HIV						
j) HIV+ patients tend to have numerous sexual partners.						
k) I believe I have the right to refuse to treat HIV+ patients if I feel uncomfortable.						
l) I would rather not come into physical contact with HIV+ patients.						
m) I would want to wear two sets of gloves when examining HIV+ patients.						
n) I believe I have the right to refuse to treat HIV+ patients to protect myself.						
o) I would be comfortable working alongside another health care provider who has HIV.						
p) I think many HIV+ patients likely have substance abuse problems.						
q) I believe I have the right to refuse to treat HIV+ patients if I am concerned about legal liability.						
r) I would rather see an HIV-negative patient than see an HIV+ patient with non-HIV-related concerns.						

s) HIV+ patients should accept responsibility for acquiring the virus.						
t) I worry about contracting HIV from HIV+ patients.						
u) I often think HIV+ patients have caused their own health problems.						
v) HIV+ patients make me uncomfortable.						
w) I would be hesitant to send HIV+ patients to get bloodwork done due to my fear of others' safety.						
x) It is a little scary to think I have touched HIV+ patients.						
y) I worry that universal precautions are not good enough to protect me from HIV+ patients.						
z) I would feel uncomfortable knowing one of my colleagues is HIV+.						
aa) HIV+ patients who have acquired HIV through injection drug use are more at fault for contracting HIV than HIV+ patients who have acquired HIV through a blood transfusion.						
bb) I tend to think that HIV+ patients do not share						

the same values as me.						
cc) HIV+ patients who have acquired HIV through sex are more at fault for contracting HIV than HIV+ patients who have acquired HIV through a blood transfusion.						
dd) It would be hard to react calmly if a patient tells me he or she is HIV+.						

32. Please enter your email address here. We will retain your email address and unique ID code in an electronic file under password protection on a secure server behind a firewall. Your email address will only be used to contact you about a follow-up survey, and in case you need to contact us to recall your unique ID code. This email address will also be used to email you your $50 electronic gift card.

Thank you for taking the time to complete our survey. We appreciate your time.
