# Supplementary material for: Decentralizing PrEP delivery: Implementation and dissemination strategies to increase PrEP uptake among MSM in Toronto, Canada
Source: PLoS One. 2021 Mar 18;16(3):e0248626. doi: 10.1371/journal.pone.0248626 (PMC7971529; doi:10.1371/journal.pone.0248626)
Supplement: S4 File — (PDF) [file pone.0248626.s004.pdf]

APPENDIX I: Decentralizing PrEP Delivery Physician Followup Questionnaire

INSTRUCTIONS: Please fill out this questionnaire to the best of your ability. Thank you very much for taking the time to participate in this study. The first few questions are about you and your practice.

1. Please enter the unique ID code on the paper or e-card you used to access the module: _______

2. Did you complete the online CME module?
   - ☐ Yes
   - ☐ No

3. In the past six months (since completing your original survey), have you completed any other CME activities about HIV PrEP?
   - ☐ Yes
   - ☐ No
   - ☐ Unsure/cannot recall

3a POPUP if 3=yes: What were these activities?
- ☐ Conference
- ☐ Online modules
- ☐ Journal articles
- ☐ Workshops/symposia
- ☐ Other:

4. Did you prescribe PrEP to the patient who gave you the card about the online PrEP CME module? (The remaining questions will refer to this individual as your "index patient").
   - ☐ Yes
   - ☐ No

4a POPUP if 4=yes What are some of the reasons that you prescribed it? (Check all that apply)
- ☐ I assessed that the patient was at increased risk of HIV infection
- ☐ I wanted to gain more experience with PrEP
- ☐ I wanted to build my therapeutic relationship with this patient
- ☐ Other (specify):

4b POPUP if yes: Please indicate if you involved, or referred to, any of the following allied personnel in the care of this index patient for whom you prescribed PrEP:

Check	Personnel	Please describe what role(s) this person played in the patient's care	In total, how many hours of their time was required in the past 6 months?
	Nurse		

	Nurse practitioner		
	Physician assistant		
	Social worker		
	Clinic pharmacist		
	Other (specify):		
	No other personnel		

4c POPUP if yes: Who draws the bloodwork for your patient?

- ☐ A commercial lab
- ☐ An onsite (i.e. hospital) lab
- ☐ Me (physician)
- ☐ Non-physician staff in my clinic (if yes, please include this in your answer to 4b above)

4d POPUP if yes Please indicate which of these activities you have conducted with this patient so far by placing a checkmark in each applicable box. Do not include tests done prior to the patient's initial request for PrEP.

**Lab tests**	**Screening**	**Baseline**	**30 days**	**Q3 mo**
**HIV Serology**				
**Hepatitis A screen**				
**Hepatitis B screen**[a]				
**Hepatitis C screen**				
**Syphilis test**				
**Gonorrhea, chlamydia urine tests**				
**Gonorrhea, chlamydia throat swabs**				
**Gonorrhea, chlamydia rectal swabs**				
**Complete Blood Count**				
**Liver transaminases**				
**Creatinine**				
**Urinalysis**				
**Medication adherence counseling**				
**Risk reduction counseling**				
**Other (specify)**				
**Other (specify)**				

**Other (specify)**				

4e) For each of the following PrEP-related visits, please indicate what OHIP billing code(s) you use/used, and how much of your time was required for the visit:

Visit	OHIP billing code(s)	Time required (minutes)
Screening		
Baseline		
30 day follow-up		
3 monthly follow-up		

4f) Please describe and quantify any additional costs incurred by your practice associated with your prescribing PrEP. (e.g. nursing costs, phlebotomy costs)

Item	Approximate cost

4g POPUP IF 4= NO: What are some of the reasons that you did not prescribe it? (Check all that apply)

- ☐ I assessed that the patient was not at increased risk of HIV infection
- ☐ The patient had a contraindication to PrEP
- ☐ The patient did not return for follow-up
- ☐ The patient did not have adequate drug coverage
- ☐ I did not feel I had enough knowledge to safely prescribe PrEP
- ☐ I do not think it is appropriate for family physicians to prescribe PrEP
- ☐ I do not have time to prescribe PrEP
- ☐ Other (specify):________

5. How do you think your experience receiving the information card from the index patient and discussing PrEP with the patient (if applicable) impacted on the quality of your doctor-patient relationship?

- ☐ Worsened the relationship a great deal
- ☐ Worsened the relationship somewhat
- ☐ No change
- ☐ Improved the relationship somewhat
- ☐ Improved the relationship a great deal

5a) If 5=1,2,4,5 In what ways has your relationship changed? (free text)

6. In the past six months (since completing your original survey), about how many other patients in each of the following risk categories have ASKED YOU about pre-exposure prophylaxis (PrEP), in addition to the index patient?

Men who have sex with men __
Heterosexual women __
Heterosexual men __
Transgender men __
Transgender women __
People in serodiscordant relationships (where 1 partner is HIV-negative and 1 is HIV-positive)__
People who inject drugs __
Other, please specify: __

7. In the past six months (since completing your original survey), to how many other patients in each of the following categories have you PRESCRIBED pre-exposure prophylaxis (PrEP), in addition to the index patient?

Men who have sex with men
Heterosexual women
Heterosexual men
Transgender men
Transgender women
People in serodiscordant relationships (where 1 partner is HIV-negative and 1 is HIV-positive)
People who inject drugs
Other, please specify:

8. How would you rate your current level of comfort prescribing PrEP?

- ☐ 1 Not comfortable at all
- ☐ 2
- ☐ 3 Somewhat comfortable
- ☐ 4
- ☐ 5 Very comfortable

9. Based on what you know currently, is your attitude towards PrEP generally:

- ☐ Very Unenthusiastic – I do not feel PrEP is an appropriate means of preventing HIV
- ☐ Unenthusiastic
- ☐ Neutral – I feel neutral about the utility of PrEP for HIV prevention

- ☐ Enthusiastic
- ☐ Very enthusiastic- I think PrEP is an essential tool in HIV prevention and am eager to prescribe it to appropriate patients.

10. In what kinds of settings do you feel PrEP could be prescribed? (check all that apply)

- ☐ Dedicated Public Health / STI clinics
- ☐ HIV specialty clinics
- ☐ Individual family physicians' offices (like any other health issue)
- ☐ Other, please specify:

11. What are current barriers to family physicians prescribing PrEP? (check all that apply)

- ☐ Family physicians are not familiar enough with PrEP to prescribe it
- ☐ It is unclear which patients should use PrEP
- ☐ Family physicians do not have enough time to assess patients to see if they would be candidates for PrEP
- ☐ Family physicians do not have enough time to prescribe PrEP
- ☐ Family physicians do not have enough time to monitor patients on PrEP appropriately
- ☐ Family physicians are not remunerated appropriately for PrEP provision
- ☐ Most patients do not have drug coverage for PrEP
- ☐ Other, please specify:

12. What supports would your practice need to assist in your ability to prescribe PrEP? (check all that apply)

- ☐ Social work support to help patients obtain drug coverage
- ☐ Continuing medical education on new research about PrEP
- ☐ Nursing support for ongoing patient counselling,
- ☐ Nursing support for routine STI and HIV testing and monitoring for adverse effects
- ☐ Other (please be as specific as possible)

13. The last few questions are about HIV more broadly. Below is a list of perceptions about persons living with HIV (PLHIV). Some of the ideas may be true for you, and some of them may not. People hold a wide range of ideas about HIV+ patients, and we are interested in your particular perceptions. Please answer the questions honestly – your responses are completely deidentified.

	Strongly Disagree	Disagree	Somewhat disagree	Somewhat Agree	Agree	Strongly Agree
a) I believe most HIV+ patients acquired the virus through risky behaviour.						
b) I think HIV+ patients have engaged in risky activities despite knowing these risks.						
c) I believe I have the right to refuse to treat HIV+ patients for the safety of other patients.						
d) I think people would not get HIV if they had sex with fewer people.						
e) HIV+ patients present a threat to my health.						
f) HIV+ patients present a threat to the health of other patients.						
g) I believe I have the right to refuse to treat HIV+ patients if other staff members are concerned about safety.						
h) I would avoid conducting certain procedures on HIV+ patients.						
i) I think if people act responsibly they will not contract HIV						
j) HIV+ patients tend to have numerous sexual partners.						
k) I believe I have the right						

to refuse to treat HIV+ patients if I feel uncomfortable.						
l) I would rather not come into physical contact with HIV+ patients.						
m) I would want to wear two sets of gloves when examining HIV+ patients.						
n) I believe I have the right to refuse to treat HIV+ patients to protect myself.						
o) I would be comfortable working alongside another health care provider who has HIV.						
p) I think many HIV+ patients likely have substance abuse problems.						
q) I believe I have the right to refuse to treat HIV+ patients if I am concerned about legal liability.						
r) I would rather see an HIV-negative patient than see an HIV+ patient with non-HIV-related concerns.						
s) HIV+ patients should accept responsibility for acquiring the virus.						
t) I worry about contracting HIV from HIV+ patients.						
u) I often think HIV+ patients have caused their own health problems.						
v) HIV+ patients make me						

uncomfortable.						
w) I would be hesitant to send HIV+ patients to get bloodwork done due to my fear of others' safety.						
x) It is a little scary to think I have touched HIV+ patients.						
y) I worry that universal precautions are not good enough to protect me from HIV+ patients.						
z) I would feel uncomfortable knowing one of my colleagues is HIV+.						
aa) HIV+ patients who have acquired HIV through injection drug use are more at fault for contracting HIV than HIV+ patients who have acquired HIV through a blood transfusion.						
bb) I tend to think that HIV+ patients do not share the same values as me.						
cc) HIV+ patients who have acquired HIV through sex are more at fault for contracting HIV than HIV+ patients who have acquired HIV through a blood transfusion.						
dd) It would be hard to react calmly if a patient tells me he or she is HIV+.						

Thank you for taking the time to complete our survey. We appreciate your time.
